# Evaluation of the Effectiveness of the Policy of Holding the Second Dose of Vaccination: Lessons from the Outbreak in Ho Chi Minh City

**DOI:** 10.3390/vaccines11020293

**Published:** 2023-01-29

**Authors:** Vu Thi Thu Trang, Le Van Truong, Truong Van Dat, Randa Elsheikh, Nguyen Tuan Anh, Dang Xuan Thang, Vo Viet Thang, Abdelrahman M. Makram, Nguyen Tien Huy, Nagasaki University Collaborative

**Affiliations:** 1 National Hospital of Traditional Medicine, Hanoi 100000, Vietnam; 2 Online Research Club, Nagasaki 852-8523, Japan; 3 Traditional Medicine Hospital, Ministry of Public Security, Hanoi 100000, Vietnam; 4 Center for Education Research and Development EdLab Asia, Hanoi 100000, Vietnam; 5 Faculty of Medicine, University of Medicine and Pharmacy, Ho Chi Minh City 700000, Vietnam; 6 Deanery of Biomedical Sciences, Edinburgh Medical School, University of Edinburgh, Edinburgh EH8 9YL, UK; 7 Faculty of Medicine and Health Sciences, Vietnam University of Traditional Medicine, Hanoi 100000, Vietnam; 8 Faculty of Medicine, College of Medicine and Pharmacy, Duy Tan University, Da Nang 550000, Vietnam; 9 Institute for Research and Training in Medicine, Biology and Pharmacy, Day Tan University, Da Nang 550000, Vietnam; 10 School of Public Health, Imperial College London, London SW7 2BX, UK; 11 School of Tropical Medicine and Global Health, Nagasaki University, Nagasaki 852-8521, Japan

**Keywords:** COVID-19, low- and middle-income countries, Vietnam, vaccination, health policy, holding the second vaccine dose

## Abstract

**Highlights:**

- Many low-and-middle-income countries (LMICs) are facing massive delays in the administration of the second and even the first doses of the COVID-19 vaccines
- However, the delays in the administration of the second dose could help governments of LMICs administer the first dose of the vaccine on a larger scale.
- A more than 21-day delay in the administration of the second dose may further improve the efficacy of the first dose.
- A longer interval between vaccination and COVID-19 is associated with improved saturation of peripheral oxygen (SPO_2_) rates, indicating that a second dose delay can help lower ICU admissions and mortality rates.

**Abstract:**

The coronavirus disease 2019 (COVID-19) pandemic has caused a lot of ethical controversy in the equal provision of healthcare, including vaccination. Therefore, our study was designed to assess the impact of Ho Chi Minh City’s policy to hold the second dose of the COVID-19 vaccine. Using a cross-sectional study design to assess low saturation of peripheral oxygen (SPO2) risk based on vaccination status, we included patients who were confirmed to have SARS-CoV-2 and were treated at home. The stepwise method was used to determine participants’ low SPO2 risk-related factors. The average age of the 2836 respondents was 46.43 ± 17.33 (years). Research results have shown that seven factors are related to the low SPO2 status of participants, including age, sneezing, shortness of breath, coughing, and fainting as COVID-19 symptoms, the number of people living with COVID-19, and a history of lung disease. A statistically significant (*p* = 0.032) finding in this study was that fully vaccinated patients had a 6% lower risk of low SPO2 compared to the first dose less than 21 days group. This result was similar in the vaccine holder group (*p* < 0.001). Holding the second dose of the COVID-19 vaccine is associated with a lower SPO2 risk than that of fully vaccinated patients. Therefore, this approach should be considered by governments as it could bring a greater benefit to the community.

## 1. Introduction

Coronavirus disease 2019 (COVID-19) is a respiratory disease caused by severe acute respiratory syndrome coronavirus 2 (SARS-CoV-2). The disease was first identified in December 2019 in Wuhan, China [1]. It then spread rapidly on a global scale and, as of June 2022, there were nearly 600 million confirmed cases and over 6 million deaths [2]. To overcome the global burden of this devastating pandemic, rapid transmission control actions were widely implemented [3] and vaccines were developed with international collaborations [4]. Most studies suggest that vaccines are still effective against circulating variants, and possibly against severe disease and death [5]. However, how long vaccine-induced immunity lasts and how transmissibility has been affected by the vaccine are still unanswered questions [5,6].

The situation in low- and middle-income countries (LMICs) may be somewhat different from their counterparts (middle- and higher-income countries) due to various reasons. These include different incremental cost-effectiveness ratios of various vaccine types in different situations [7], vaccine hesitancy or low acceptance rates [8], the usage of vaccines with lower efficacy [9], and the lower overall purchasing capacity for the vaccines [10]. In Vietnam, an LMIC, the fourth wave of the pandemic lasted for more than eight months with a total of over two million confirmed COVID-19 cases and 35,480 deaths nationwide, of which the largest contribution came from Ho Chi Minh City, with over 20,000 deaths. According to the report of the Vietnam National Steering Committee on COVID-19 prevention and control, in the fourth wave, almost 100% of COVID-19 patients were infected with the Delta variant [11]. Infection control policies such as social distancing and mandatory mask-wearing did not seem to be effective at this stage [12].

An expected solution to the problem is vaccination; however, the Vietnamese government is facing a shortage of vaccine supplies and medical staff. The solution applied by the government was to prioritize vaccines based on people’s risk factors and to administer the vaccines through a center-based policy, where people receive vaccines at larger, more crowded healthcare facilities [12]. The centralized vaccination process, however, has resulted in the exposure of uninfected individuals. Ho Chi Minh City government has applied the measure of vaccination with the first dose widely throughout the city, starting with the elderly, people who have comorbidities, and medical staff [11]. The solution has proved effective when counting the number of people having access to the COVID-19 vaccine; specifically, by the end of September 2021 in District 5, Ho Chi Minh City, 98% of people over 18 years old have received COVID-19 vaccines, of which 31% have received two doses [13]. However, this approach has generated controversy throughout implementation regarding doubts about the evidence for the benefit of a single dose of vaccine [12,14]. Therefore, our study was designed to evaluate the effectiveness of administering only one dose on the saturation of peripheral oxygen (SPO_2_) index of future COVID-19 patients treated at home.

## 2. Methods

### 2.1. Study Design, Population, and Conduction

The cross-sectional descriptive study which follows the Strengthening the Reporting of Observational Studies in Epidemiology (STROBE) statement (checklist available as Supplementary Table S1) [14] was conducted from 15 August to 15 November 2021, according to decision No. 980/UBND-VP issued on 14 August 2021 by the People’s Committee of District 5, Ho Chi Minh City, Vietnam. The main purpose of the study was to detect the severe condition of COVID-19 patients treated at home through a low SPO_2_ index (SPO_2_ ≤ 93%) [15], thus making a timely admission decision. All patients with confirmed COVID-19 being treated at home were provided a link by the medical staff to take part in a Zalo^®^ group, a WhatsApp^®^-comparable software program, after which they were provided with a questionnaire study to assess their initial status.

### 2.2. Questionnaire Design and Survey Conduction

The questionnaire consisted of two parts. The first part had ten questions about basic demographics. The second part contained 40 questions about the clinical status and the 23 symptoms of COVID-19 based on the WHO report, SPO_2_ index, highest and lowest body temperature in the last 24 h, and sneezing, which is a common finding in COVID-19 patients but not reported in the WHO list [15]. Our study divided the characteristics of COVID-19 vaccination into three groups, including the first dose less than 21 days group, which included patients who were infected with COVID-19 within 21 days following the administration of the first dose; the full vaccination group, which included patients with COVID-19 who have received the second dose at or after 21 days; and the delayed second dose group, which included COVID-19 patients who had had their first dose for more than 21 days but had not yet received their second dose. The time of 21 days after vaccination was established based on the evidence of stable immunity formation following the first and second doses of vaccine [16,17,18].

### 2.3. Data Analysis

We underwent a descriptive statistical analysis using T-test student, Chi-square, and ANOVA tests to compare demographic characteristics and clinical characteristics according to vaccination status and classification of SPO_2_ index (normal SPO_2_ and low SPO_2_). Multivariable linear regression analysis was used to evaluate factors related to the low SPO2 of patients vaccinated against COVID-19, using the stepwise AIC method on the MASS package to determine the optimal model. All analyses were performed on R language version 4.1.0.

### 2.4. Ethical Considerations

Before the conduction of this study, data were collected from the home care program for COVID-19 patients at the People’s Committee of District 5, Ho Chi Minh City (Decision No. 980/UBND-VP dated 14 August 2021). The benefits (e.g., increased knowledge about the policy impact) and probable burdens (e.g., time burden and some feelings of discomfort) were explained to all patients before the distribution of the survey. It was emphasized that there are no direct benefits to the patients, including financial incentives. It was also emphasized that patients can withdraw at any time by quitting the survey page; however, once the questionnaire has been submitted, there was no way of deleting the collected data as personal data were not collected. To ensure further confidentiality, the IP address tracking was disabled to disallow any attempt at identifying the enrolled participants. All data will be stored for five years after the publication of this manuscript.

Before moving on to the questionnaire page, electronic informed consent was obtained from all patients after reading all the details about the project. If the participant ticked “I consent to fill in the questionnaire”, they were redirected to the questionnaire page. Otherwise, a skip-logic function ended the survey.

## 3. Results

Our program had a response rate of 38.5% (2548/6616 patients), of which 56.3% were female. The average age of participants was 46.43 ± 17.33 (years), and 7.3% (186/2548) of the patients were in the full vaccination group.

Table 1 shows that the rate of asymptomatic patients in the “first dose less than 21 days”, “holding the second dose”, and “full vaccination” groups tends to increase statistically as the time from injection and number of vaccinations increases, with rates of 25.1%, 28.8%, and 39.3%, respectively. The number of symptoms with which the patients typically presented revealed a similar trend, with the average number of symptoms being 3.27 ± 3.47 (points), 3.12 ± 3.42 (points), and 1.92 ± 2.58 (points), respectively. The study results also showed that the lowest and highest temperature and the lowest SPO_2_ recorded in the last 24 h of patients who were not previously vaccinated showed statistically significantly higher values than those of the vaccinated group. Furthermore, the number of presenting symptoms in the unvaccinated group was significantly higher than in the rest of the groups, with a positive rate of 17/23 symptoms.

Table 2 shows that the group of normal SPO_2_ patients has a statistically significantly lower mean age than the low SPO_2_ group. The average time from vaccination to confirmed COVID-19 in the low SPO_2_ group was 24.33 ± 12.05 (days), which was statistically significantly lower than that of the normal SPO_2_ group, with 27.52 ± 13.65 (days). According to the medical record, the low SPO_2_ group had a statistically significantly higher rate of underlying disease (63.0%) than the normal SPO_2_ group, including hypertension, cardiovascular disease, diabetes, other lung diseases, dementia, and kidney disease.

**Figure 1:**
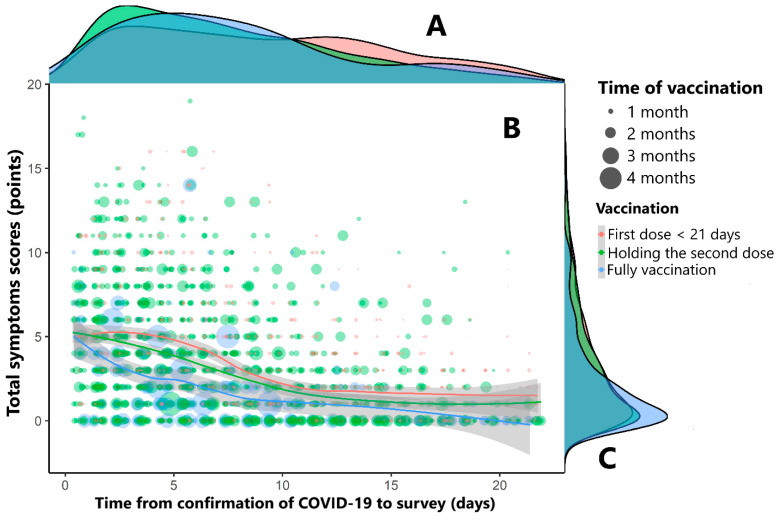
The relationship between the duration of COVID-19, the number of clinical symptoms, and vaccination type. Three colors have been used to represent the characteristics of COVID-19 patients, as follows: red represents patients with “first dose < 21 days”, green for “holding the second doses”, and blue for “fully vaccinated”. (**A**,**C**) use density histograms to show the distribution of the study subjects from the time of illness to the time of the survey, and the number of symptoms of COVID-19 patients. (**B**) uses a scatterplot to show the relationship between the duration of illness and the number of symptoms, and the size of the circle corresponds to the time between the last vaccination and the survey. Loess regression was used to draw smoothing lines according to the vaccination status of COVID-19 patients. Time of vaccination refers to the time from the last dose of the vaccine to the time of study participation.

Figure 1 shows that the total number of symptoms corresponding to patients taking the first dose less than 21 days, holding the second dose, and fully vaccinated reached the highest value on the first day after a confirmed infection with SARS-CoV-2 and tended to decrease and maintain a steady rate over the next ten days.

**Figure 2:**
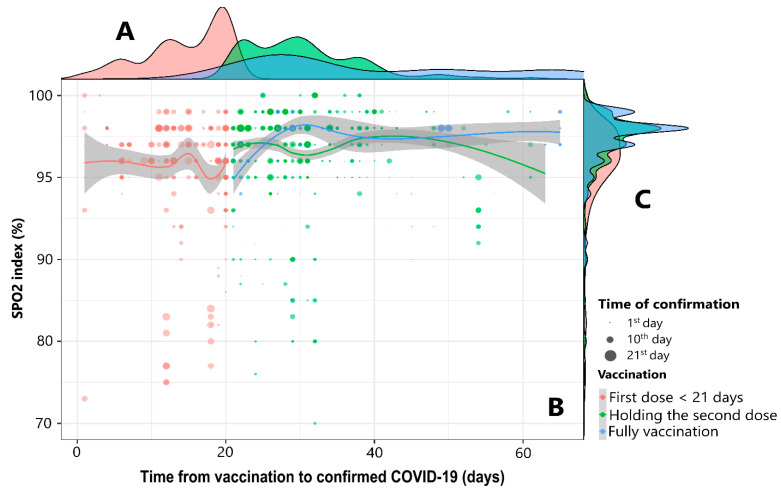
The relationship between the time from vaccination to confirmed COVID-19 and the SPO_2_ index by the time of confirmation and vaccination status. Three colors have been used to represent the characteristics of COVID-19 patients, as follows: red represents patients with “first dose < 21 days”, green for “holding the second doses”, and blue for “fully vaccinated”. (**A**,**C**) use density histograms to show the distribution of the study subjects from the time of illness to the time of the survey and the SPO_2_ index. (**B**) uses a scatterplot to show the relationship between the duration of illness and the SPO_2_ index, and the size of the circle corresponds to the time between the last vaccination and the survey. Loess regression was used to draw smoothing lines according to the SPO_2_ index of patients. The time of vaccination refers to the time from the last dose of the vaccine to the time of study participation.

Figure 2 shows that patients in the first dose less than 21 days group had an average SPO_2_ value of 95.4 ± 4.3%, the index of holding the second dose group was 96.8 ± 3.0%, and the full vaccination group had an index of 97.4 ± 1.6%. The group of patients with a first dose from 21 days to 50 days and a full vaccine from 21 days to 65 days showed a stable SPO_2_ index above 97%.

Table 3 shows that a ten-year increase in patient age was associated with a 2% (95% CI: 1–3%, *p* < 0.001) increased risk of low SPO_2_. Each increase in cohabitation was associated with a 1% (95% CI: 0–2%, *p* = 0.025) increase in the risk of low SPO_2_. Patients with a history of lung diseases were associated with a 35% (95% CI: 21–51%, *p* < 0.001) increased risk of low SPO_2_. The female gender was associated with a 5% (95% CI: 2–7%, *p* = 0.002) reduction in the risk of low SPO_2_. Positive symptoms including shortness of breath, sneezing, fainting, and cough were significantly associated with an increased risk of low SPO_2_ of 30%, 10%, 8%, and 5%, respectively. Patients infected with SARS-CoV-2, including both the first dose of less than 21 days and full vaccination groups, were statistically significantly associated with a 6% reduction in the risk of low SPO_2_. The above model recorded a good forecast of the low SPO_2_ situation, expressed through the area under the curve (AUC) reaching 86.4%. Supplementary Table S2 presents the same models along with a comparison between males and females with respect to the other covariates.

## 4. Discussion

Our study aimed to determine the factors associated with low SPO_2_ in Vietnamese post-vaccination COVID-19 patients treated at home to evaluate the effects of delaying the second dose or administering only one dose of vaccination. Through a multivariable logistic regression model, nine factors that affected COVID-19 patients’ SPO_2_ index were identified. A 10-year increase in age, developing sneezing, shortness of breath, coughing, and fainting as COVID-19 symptoms, co-living with an additional one person above the reported average, and having a history of lung disease were all associated with a higher risk of developing low SPO_2_. Contrastingly, COVID-19 patients belonging to the group of stable immunity (full vaccination group or holding second dose group) and female gender were found to be linked to a lower risk of developing low SPO_2_. Furthermore, vaccination was associated with an increased proportion of asymptomatic patients, with 15.3%, 25.1%, 28.8%, and 39.3% asymptomatic rates in patients who did not receive any dose, first dose less than 21 days, holding the second dose, and full vaccination, respectively.

In 2021, Moghadas et al. evaluated the optimal time for the administration of the second dose of the Moderna and Pfizer-BioNTech vaccines. They found a significant reduction in infection, hospitalization, and death rates when the second dose was deferred 12–15 weeks from the first dose less than 21 days [20]. Similarly, Silva et al. studied the ideal delay between COVID-19 dose administration and its effect on ICU admission rates. As in our study, they reported that a minimum of a 4-week delay in second dose administration is expected to decrease ICU admission rates, as it gives time to the first dose less than 21 days to achieve a higher efficacy [21]. An Oxford study carried out to assess the reactogenicity and the immunogenicity following the delay in the administration of the ChAdOx1 nCoV-19 included participants with 8–12, 15–25, and 44–45-week intervals between the doses. Antibody levels measured 6 months (median = 3738, IQR = 1824–6625) after the administration of the second dose were found to be significantly higher (*p* < 0.001) than those with a 15–25-week interval between the first and second doses (median = 1860, IQR = 917–4992), showing that the delay in the administration of the second dose of AstraZeneca vaccine increases its efficacy [22].

Regarding the factors associated with worse COVID-19 outcomes, a systematic review was conducted to determine the factors associated with higher mortality risk following SARS-CoV-2 infection. Consistent with our study, old age, male gender, and previous lung diseases had a positive correlation with COVID-19 mortality. A prolonged inflammatory response following the weakening of immunity and the excessive release of type 2 cytokines was found to be the reason behind worse outcomes in elderly patients [23]. Moreover, hypoxia in COVID-19 patients is associated with viral lung injury, with alternating regions of hyperventilation and hypoventilation [24]. Therefore, sneezing influences airflow and ventilation pressure change [25], which may be responsible for the more common occurrence of low SPO_2_ in this group of patients.

Our findings suggest that several benefits can be obtained from holding the second dose of vaccination, which are comparable to the benefits seen in the full vaccination group. Achieving the full effectiveness of the first dose in less than 21 days was shown to be associated with higher asymptomatic rates, and less severe outcomes. A longer interval between vaccination and COVID-19 infection was further associated with improved SPO_2_ rates, which indicates that a second dose delay could contribute to lowering ICU admissions and mortality rates. However, our findings should be cautiously interpreted on a global scale, due to various reasons including the higher use rate of AstraZeneca and the lower BMI when compared to higher-income countries, as well as the statistically significant differences between the two groups, detected in Table 2.

Although, as far as we know, this is the only study investigating the delay in the administration of COVID-19 vaccines in Vietnam, better results could have been obtained by investigating individual vaccines rather than unspecified vaccines as was carried out by Payne et al. in the UK [26]. As for the analysis of individual COVID-19 vaccines, a study in Canada by Hall et al. reported that extending the interval between the two doses of the Pfizer-BioNTech vaccine from 3–6 weeks to 8–16 weeks leads to a better antibody response in female healthcare workers [27,28]. The same results were reported earlier for the AstraZeneca vaccine for both the second and the booster dose [22]. However, we were not able to do so due to the limited variability of sample sizes for each vaccine type. Another limitation of this study is the study design, which did not allow for a follow-up. Moreover, we were not able to assess more detrimental clinical features and outcomes, choosing to analyze only the SPO_2_. Lastly, it is important to consider the findings of this study in the context of wider public health in Vietnam, where nearly a quarter of the population is hesitant to receive the vaccine or offer it to their children [29,30]. Moreover, people with medical or allergic history are more likely to decline vaccination, further contributing to the health inequalities gap related to vaccination [29].

## 5. Conclusions

The study shows that holding the second dose of vaccination against COVID-19 is as effective as obtaining a full vaccination in terms of increasing the asymptomatic rate and reducing the rate of low SPO_2_. Moreover, the SPO_2_ rate was different in the unvaccinated patients and the group receiving the first dose of the vaccine less than 21 days following SARS-CoV-2 infection. Our study provides further evidence for policymakers about how vaccine distribution ensures maximum protection for the community in the face of limited vaccine supply. However, the study also suggests the need for policies to limit the process of cross-contamination during vaccination, helping to improve the effectiveness of patient protection and avoid outbreaks related to an infection at the vaccination site. Although it has not been given sufficient attention yet, sneezing was present in 21.4% of our sample and was associated with a 10% increased risk of low SPO_2_ together with an increased risk of disease transmission.

## Figures and Tables

**Table 1:** Baseline patients’ characteristics according to their vaccination status.

Characteristics	Total	First Dose Less Than 21 Days	Holding the Second Dose	Full Vaccination	*p*-Value
	N = 2548	N = 905	N = 1457	N = 186	
Type of vaccination					<0.001 *
AstraZeneca	2202 (86.42%)	695 (76.80%)	1329 (91.21%)	178 (95.70%)	
Moderna	286 (11.22%)	177 (19.56%)	108 (7.41%)	1 (0.54%)	
Pfizer	33 (1.30%)	22 (2.43%)	9 (0.62%)	2 (1.08%)	
Not specified	27 (1.06%)	11 (1.22%)	11 (0.75%)	5 (2.69%)	
Total symptom scores (points)	3.09 (3.40)	3.27 (3.47)	3.12 (3.42)	1.92 (2.58)	<0.001 *
Day of infection (days)	8.11 (5.35)	9.21 (5.70)	7.45 (5.02)	7.86 (5.27)	<0.001 *
Asymptomatic					<0.001 *
No	719 (28.22%)	227 (25.08%)	419 (28.76%)	73 (39.25%)	
Yes	1829 (71.78%)	678 (74.92%)	1038 (71.24%)	113 (60.75%)	
Highest temperature	36.79 (0.66)	36.86 (0.77)	36.77 (0.60)	36.63 (0.55)	0.001 *
Lowest temperature	36.34 (0.48)	36.36 (0.50)	36.33 (0.47)	36.30 (0.50)	0.186
Lowest SPO_2_	96.48 (3.36)	95.39 (4.27)	96.77 (3.03)	97.38 (1.63)	<0.001 *
Cough (Yes)	1064 (41.76%)	400 (44.20%)	596 (40.91%)	68 (36.56%)	0.094
Not eating well (Yes)	745 (29.24%)	303 (33.48%)	402 (27.59%)	40 (21.51%)	0.001 *
Stuffy nose (Yes)	703 (27.59%)	219 (24.20%)	450 (30.89%)	34 (18.28%)	<0.001 *
Decrease/loss of smell (Yes)	614 (24.10%)	209 (23.09%)	373 (25.60%)	32 (17.20%)	0.028 *
Fatigue (Yes)	517 (20.29%)	207 (22.87%)	295 (20.25%)	15 (8.06%)	<0.001 *
Insomnia (Yes)	466 (18.29%)	174 (19.23%)	276 (18.94%)	16 (8.60%)	0.002 *
Decrease/loss of taste (Yes)	435 (17.07%)	158 (17.46%)	249 (17.09%)	28 (15.05%)	0.729
Sore throat (Yes)	424 (16.64%)	136 (15.03%)	270 (18.53%)	18 (9.68%)	0.003 *
Muscle pain (Yes)	392 (15.38%)	163 (18.01%)	219 (15.03%)	10 (5.38%)	<0.001 *
Runny nose (Yes)	390 (15.31%)	150 (16.57%)	216 (14.82%)	24 (12.90%)	0.331
Headache (Yes)	369 (14.48%)	145 (16.02%)	213 (14.62%)	11 (5.91%)	0.002 *
Diarrhea (Yes)	322 (12.64%)	155 (17.13%)	161 (11.05%)	6 (3.23%)	<0.001 *
Chills (Yes)	308 (12.09%)	137 (15.14%)	159 (10.91%)	12 (6.45%)	<0.001 *
Dizziness (Yes)	224 (8.79%)	100 (11.05%)	118 (8.10%)	6 (3.23%)	0.001 *
Joint pain (Yes)	220 (8.63%)	119 (13.15%)	97 (6.66%)	4 (2.15%)	<0.001 *
Fever (Yes)	212 (8.32%)	100 (11.05%)	102 (7.00%)	10 (5.38%)	0.001 *
Shortness of breath (Yes)	163 (6.40%)	61 (6.74%)	98 (6.73%)	4 (2.15%)	0.049 *
Chest pain (Yes)	145 (5.69%)	46 (5.08%)	94 (6.45%)	5 (2.69%)	0.070
Red eyes (Yes)	115 (4.51%)	39 (4.31%)	69 (4.74%)	7 (3.76%)	0.780
Faint (Yes)	109 (4.30%)	48 (5.33%)	60 (4.15%)	1 (0.54%)	0.012 *
Nausea/vomiting (Yes)	75 (2.94%)	35 (3.87%)	35 (2.40%)	5 (2.69%)	0.120
Rash on skin (Yes)	59 (2.32%)	21 (2.32%)	36 (2.47%)	2 (1.08%)	0.582
Loss of speech (Yes)	27 (1.06%)	7 (0.77%)	19 (1.30%)	1 (0.54%)	0.478
Sneeze (Yes) ^†^	540 (21.19%)	179 (19.78%)	324 (22.24%)	37 (19.89%)	0.329

Abbreviations: NA (not applicable). ^†^ Sneezing is a common clinical symptom but has not been reported as a symptom of COVID-19 according to WHO. * Indicates a statistically significant *p*-value of less than 0.05. The statistical tests used include ANOVA, chi-square, and Phi and Cramer’s V.

**Table 2:** Baseline characteristics of the included patients according to the SPO_2_ status.

Characteristics	Total	SPO_2_ > 93%	SPO_2_ ≤ 93%	*p*-Value
	N = 1173	N = 1081	N = 92	
Age (years)	45.21 (16.04)	44.40 (15.73)	54.77 (16.70)	<0.001 *
Gender				0.110
Male	463 (39.47%)	419 (38.76%)	44 (47.83%)	
Female	710 (60.53%)	662 (61.24%)	48 (52.17%)	
BMI (kg/m^2^)	23.14 (3.73)	23.17 (3.78)	22.81 (3.16)	0.307
Time from vaccination to confirmed COVID-19 (days)	27.28 (13.56)	27.52 (13.65)	24.33 (12.05)	0.021 *
Time from confirmed COVID-19 to survey (days)	9.09 (5.18)	9.08 (5.14)	9.15 (5.59)	0.911
Healthcare staff				0.100
No	1167 (99.49%)	1075 (99.44%)	92 (100.00%)	
Yes	6 (0.51%)	6 (0.56%)	0 (0.00%)	
Number of people living with COVID-19 patients	2.60 (1.62)	2.57 (1.64)	2.88 (1.27)	0.033 *
Total symptom scores (points)	2.82 (3.26)	2.58 (3.11)	5.63 (3.72)	<0.001 *
Asymptomatic				<0.001 *
No	365 (31.12%)	357 (33.02%)	8 (8.70%)	
Yes	808 (68.88%)	724 (66.98%)	84 (91.30%)	
Type of vaccination				<0.001 *
AstraZeneca	994 (84.74%)	930 (86.03%)	64 (69.57%)	
Moderna	147 (12.53%)	127 (11.75%)	20 (21.74%)	
Pfizer	24 (2.05%)	19 (1.76%)	5 (5.43%)	
Not specified	8 (0.68%)	5 (0.46%)	3 (3.26%)	
Medical record				<0.001 *
No	645 (54.99%)	611 (56.52%)	34 (36.96%)	
Yes	528 (45.01%)	470 (43.48%)	58 (63.04%)	
Hypertension (Yes)	225 (19.18%)	198 (18.32%)	27 (29.35%)	0.015 *
Cardiovascular disease (Yes)	126 (10.74%)	108 (9.99%)	18 (19.57%)	0.008 *
Diabetes (Yes)	106 (9.04%)	91 (8.42%)	15 (16.30%)	0.019 *
Dementia (Yes)	19 (1.62%)	12 (1.11%)	7 (7.61%)	<0.001 *
Kidney disease (Yes)	14 (1.19%)	10 (0.93%)	4 (4.35%)	0.019 *
Other lung disease (Yes)	14 (1.19%)	4 (0.37%)	10 (10.87%)	<0.001 *
Obesity (Yes) ^†^	258 (21.99%)	245 (22.66%)	13 (14.13%)	0.077
Systemic disease (Yes)	69 (5.88%)	62 (5.74%)	7 (7.61%)	0.615
Cancer (Yes)	33 (2.81%)	28 (2.59%)	5 (5.43%)	0.175
Liver disease (Yes)	24 (2.05%)	24 (2.22%)	0 (0.00%)	0.250
Asthma (Yes)	11 (0.94%)	10 (0.93%)	1 (1.09%)	0.594
COPD (Yes)	8 (0.68%)	8 (0.74%)	0 (0.00%)	1.000
Organ transplants (Yes)	3 (0.26%)	3 (0.28%)	0 (0.00%)	1.000
Down syndrome (Yes)	3 (0.26%)	3 (0.28%)	0 (0.00%)	1.000
HIV/AIDS (Yes)	3 (0.26%)	3 (0.28%)	0 (0.00%)	1.000
Sickle cell anemia (Yes)	3 (0.26%)	3 (0.28%)	0 (0.00%)	1.000
Addiction (Yes)	3 (0.26%)	3 (0.28%)	0 (0.00%)	1.000

Abbreviations: BMI (body mass index); COPD (chronic obstructive lung disease); HIV/AIDS (human immunodeficiency virus/acute immunodeficiency syndrome). ^†^ Obesity is defined according to the Western Pacific region standards, with a body mass index (BMI) of ≥25 kg/m^2^ [19]. * Indicates a statistically significant *p*-value of less than 0.05. The statistical tests used include T-test, chi-square, and Phi and Cramer’s V.

**Table 3:** Univariable and multivariable logistic regression model to predict low SPO_2_.

	Univariable			Multivariable		
Predictors	Estimates	95% CI	*p*-Value	Estimates	95% CI	*p*-Value
(Intercept)	–	–	–	0.96	0.91–1.02	0.237
Age (each 10 years)	1.03	1.02–1.04	<0.001 *	1.02	1.01–1.03	<0.001 *
Number of people living with COVID-19 patients in the same household	1.01	1.00–1.02	0.02 *	1.01	1.00–1.02	0.025 *
Lung diseases ^#^						
No	Reference			Reference		
Yes	1.54	1.38–1.73	<0.001 *	1.35	1.21–1.51	<0.001 *
Gender						
Male	Reference			Reference		
Female	0.97	0.94–1.00	0.028 *	0.95	0.93–0.98	0.002 *
Shortness of breath						
No	Reference			Reference		
Yes	1.37	1.28–1.46	<0.001 *	1.30	1.22–1.38	<0.001 *
Sneeze						
No	Reference			Reference		
Yes	1.16	1.11–1.20	<0.001 *	1.10	1.06–1.15	<0.001 *
Fainting						
No	Reference			Reference		
Yes	1.12	1.05–1.20	0.001 *	1.08	1.02–1.15	0.014 *
Cough						
No	Reference			Reference		
Yes	1.12	1.09–1.15	<0.001 *	1.05	1.02–1.08	0.001 *
Vaccination						
First dose less than 21 days	Reference			Reference		
Holding the second dose *	0.94	0.91–0.97	0.001 *	0.94	0.91–0.97	<0.001 *
Full vaccination	0.91	0.85–0.97	0.003 *	0.94	0.88–0.99	0.032 *
Observations		1142		1142		
R^2^				0.186		
AUC				86.4%		

* The holding the second dose group includes COVID-19 patients who have had their 1st dose for more than 21 days but have not received their 2nd dose. ^#^ Lung diseases included chronic obstructive lung disease, asthma, and other lung diseases. * Indicates a statistically significant *p*-value of less than 0.05.

## Data Availability

All the data used in this study are available upon contacting the corresponding author (Nguyen Tien Huy) at tienhuy@nagasaki-u.ac.jp.

## Supplementary Materials

The following supporting information can be downloaded at: https://www.mdpi.com/article/10.3390/vaccines11020293/s1, Supplementary Table S1: STROBE Statement—Checklist of items that should be included in reports of cross-sectional studies, Supplementary Table S2: Univariable and multivariable logistic regression model to predict low SPO_2_, sub-grouped by gender.

## The Nagasaki University Collaborative

Included the following contributing authors: Le Van Truong; Hoang Thi Tho; Le Thi Hanh; Nguyen Hong Hanh; Nguyen Thanh Nga; Nguyen Thi Kim Ngan (Traditional Medicine Hospital, Ministry of Public Security, Hanoi 100000, Vietnam); Nguyen Bao Tran; Nguyen Thi Bich Tram; Huynh Ngo Minh Trang (University of Medicine and Pharmacy at Ho Chi Minh city, Ho Chi Minh city 700000, Vietnam); Bui Thi Ngan; Phan Thi Diep; Nguyen Tien Quang (Thai Binh University of Medicine and Pharmacy, Thai Binh 410000, Vietnam); Ngo Quang Trung, Tran Danh Tien Thinh(Hai Phong University of Medicine And Pharmacy, Hai Phong 180000, Vietnam); Nguyen Thanh An (Faculty of Medicine, College of Medicine and Pharmacy, Duy Tan University, Da Nang 550000, Vietnam); Le Duc Anh Tai; Le Ta Thanh Nga; Le Thi Minh Hanh; Nguyen Hoang Minh Tam; Nguyen Thi Hang Vi; Nguyen Thi Minh Hang; Nguyen Thuy Quynh; Nguyen Van Tuan; To Cat Tien; Vo Viet Thang (Duy Tan University, Danang 550000, Vietnam); Le Ngoc Huynh; Pham Thi Khanh Doan; Hoang Phuc An; Nguyen Thi Ngoc Tran (Cantho University of Medicine and Pharmacy, Cantho 900000, Vietnam); Thoa Le (Cardiovascular Research Labs, Methodist Hospital, Merrillville, IN 46410, USA); Dong Thanh Thien (Duc Giang General Hospital, Hanoi 100000, Vietnam); Tran Thi My Nhan (Genesolutions Company, Hanoi 100000, Vietnam); Nguyen Ha My (Hanoi Hospital of Traditional Medical, Hanoi 100000, Vietnam); Hoang Thi Hai Yen (Hanoi Medical university, Hanoi 100000, Vietnam); Nguyen Khoi Quan (VinUniversity, Hanoi 100000, Vietnam); Nguyen Thi Tinh; Tran Thi Kieu Oanh (Hue Medicine University, Hue 530000, Vietnam); Nguyen Thi Lan Phuong (National Hospital of Dermatology and Venereology, Hanoi 100000, Vietnam); Vu Thi Thu Trang (National of traditional medicine Hospital, Hanoi 100000, Vietnam); Nguyen Thi Nguyen (Obstetrics and Pediatrics Hospital, Vinh Phuc 280000, Vietnam); Ha Xuan Nam; Huynh Thi Diem Thuy (Online Research Club); Thi Viet Nga (Pham Ngoc Thach University of Medicine, Ho Chi Minh City 700000, Vietnam); Nguyen Thi Kim Hien (Pharmacity Pharmacy, Hanoi 100000, Vietnam); Ngo Thi Phuong (Traditional Medicine and Rehabilitation Hospital, Bac Ninh 220000, Vietnam); Tran Quoc Huy (Traditional Medicine Hospital, Vinh Phuc 280000, Vietnam); Nguyen Van Dieu (Vietnam national cancer Hospital, Hanoi 100000, Vietnam); Nguyen Thi Ngoc Thuy; Phan Dinh An; Vu Thi Tho; Nguyen Anh Tuan (Vietnam University Of Traditional Medicine, Hanoi 100000, Vietnam); Ngo Thi Thanh Tu (Vinmec Times City Hospital, Hanoi 100000, Vietnam); Pham Thi Huynh Anh (Vo Truong Toan University, Hau Giang 910000, Vietnam); Trinh Thi Phuong (Xuan Mai Vaccium center, Hanoi 100000, Vietnam).

## Abbreviations

**ANOVA**	analysis of variance
**BMI**	body mass index
**COPD**	chronic obstructive lung disease
**COVID-19**	coronavirus disease 2019
**HIV/AIDS**	human immunodeficiency virus/acute immunodeficiency syndrome
**LMICs**	low- and middle-income countries
**SARS-CoV-2**	Severe Acute Respiratory Syndrome Coronavirus 2
**SPO_2_**	saturation of peripheral oxygen
