# Supplementary material for: Evaluation of the Effectiveness of the Policy of Holding the Second Dose of Vaccination: Lessons from the Outbreak in Ho Chi Minh City
Source: Vaccines (Basel). 2023 Jan 29;11(2):293. doi: 10.3390/vaccines11020293 (PMC9967005; doi:10.3390/vaccines11020293)
Supplement: Supplementary file 1 [file vaccines-11-00293-s001.zip › vaccines-2127687-supplementary.pdf]

Evaluation of the effectiveness of the policy of delaying the second dose of vaccination: Lessons from the outbreak in Ho Chi Minh city

Supplementary Table S1. STROBE Statement — Checklist of items that should be included in reports of ***cross-sectional studies***.

	Item No	Recommendation	Page No
**Title and abstract**	1	(*a*) Indicate the study's design with a commonly used term in the title or the abstract	1, 2
		(*b*) Provide in the abstract an informative and balanced summary of what was done and what was found	1, 2
**Introduction**			
Background/rationale	2	Explain the scientific background and rationale for the investigation being reported	2
Objectives	3	State specific objectives, including any prespecified hypotheses	2
**Methods**			
Study design	4	Present key elements of study design early in the paper	3
Setting	5	Describe the setting, locations, and relevant dates, including periods of recruitment, exposure, follow-up, and data collection	3
Participants	6	(*a*) Give the eligibility criteria, and the sources and methods of selection of participants	3
Variables	7	Clearly define all outcomes, exposures, predictors, potential confounders, and effect modifiers. Give diagnostic criteria, if applicable	3
Data sources/ measurement	8*	For each variable of interest, give sources of data and details of methods of assessment (measurement). Describe comparability of assessment methods if there is more than one group	3
Bias	9	Describe any efforts to address potential sources of bias	3

Study size	10	Explain how the study size was arrived at	NA
Quantitative variables	11	Explain how quantitative variables were handled in the analyses. If applicable, describe which groupings were chosen and why	3
Statistical methods	12	(*a*) Describe all statistical methods, including those used to control for confounding	3
		(*b*) Describe any methods used to examine subgroups and interactions	NA
		(*c*) Explain how missing data were addressed	NA
		(*d*) If applicable, describe analytical methods taking account of sampling strategy	NA
		(*e*) Describe any sensitivity analyses	NA
**Results**			
Participants	13*	(a) Report numbers of individuals at each stage of study—eg numbers potentially eligible, examined for eligibility, confirmed eligible, included in the study, completing follow-up, and analysed	4
		(b) Give reasons for non-participation at each stage	NA
		(c) Consider use of a flow diagram	NA
Descriptive data	14*	(a) Give characteristics of study participants (eg demographic, clinical, social) and information on exposures and potential confounders	4
		(b) Indicate number of participants with missing data for each variable of interest	NA
Outcome data	15*	Report numbers of outcome events or summary measures	4-8
Main results	16	(*a*) Give unadjusted estimates and, if applicable, confounder-adjusted estimates and their precision (eg, 95% confidence interval). Make clear which confounders were adjusted for and why they were included	8

		(*b*) Report category boundaries when continuous variables were categorized	8
		(*c*) If relevant, consider translating estimates of relative risk into absolute risk for a meaningful time period	NA
Other analyses	17	Report other analyses done—eg analyses of subgroups and interactions, and sensitivity analyses	NA
**Discussion**			
Key results	18	Summarise key results with reference to study objectives	8
Limitations	19	Discuss limitations of the study, taking into account sources of potential bias or imprecision. Discuss both direction and magnitude of any potential bias	9
Interpretation	20	Give a cautious overall interpretation of results considering objectives, limitations, multiplicity of analyses, results from similar studies, and other relevant evidence	9
Generalisability	21	Discuss the generalisability (external validity) of the study results	9
**Other information**			
Funding	22	Give the source of funding and the role of the funders for the present study and, if applicable, for the original study on which the present article is based	10

Supplementary Table S2. Univariable and multivariable logistic regression model to predict low SPO2, sub-grouped by gender.

	Univariable			Gender						Multivariable		
				Female			Male					
**Predictors**	**Estimates**	**95% CI**	**P-value**	**Estimates**	**95% CI**	**P-value**	**Estimates**	**95% CI**	**P-value**	**Estimates**	**95% CI**	**P-value**
**(Intercept)**	–	–	–	0.94	0.88 – 1.00	0.054	0.98	0.88 – 1.09	0.713	0.96	0.91 – 1.02	0.237
**Age (each 10 years)**	1.03	1.02 – 1.04	<0.001*	1.02	1.01 – 1.03	<0.001	1.03	1.01 – 1.05	0.001	1.02	1.01 – 1.03	<0.001*
**Number of people living with COVID-19 patients in the same household**	1.01	1.00 – 1.02	0.02*	1.01	0.99 – 1.02	0.331	1.01	1.00 – 1.03	0.09	1.01	1.00 – 1.02	0.025*
**Lung diseases[#]**												
No	Reference			Reference			Reference			Reference		
Yes	1.54	1.38 – 1.73	<0.001*	1.42	1.23 – 1.64	<0.001	1.17	0.98 – 1.39	0.075	1.35	1.21 – 1.51	<0.001*
**Gender**												
Male	Reference									Reference		
Female	0.97	0.94 – 1.00	0.028*							0.95	0.93 – 0.98	0.002*
**Shortness of breath**												
No	Reference			Reference			Reference			Reference		
Yes	1.37	1.28 – 1.46	<0.001*	1.33	1.23 – 1.42	<0.001	1.21	1.09 – 1.35	0.001	1.30	1.22 – 1.38	<0.001*
**Sneeze**												
No	Reference			Reference			Reference			Reference		
Yes	1.16	1.11 – 1.20	<0.001*	1.06	1.02 – 1.11	0.005	1.18	1.10 – 1.26	<0.001	1.10	1.06 – 1.15	<0.001*
**Fainting**												
No	Reference			Reference			Reference			Reference		
Yes	1.12	1.05 – 1.20	0.001*	1.18	1.09 – 1.28	<0.001	0.98	0.89 – 1.09	0.774	1.08	1.02 – 1.15	0.014*
**Cough**												
No	Reference			Reference			Reference			Reference		

Yes	1.12	1.09 – 1.15	<0.001*	1.03	0.99 – 1.06	0.166	1.12	1.05 – 1.18	<0.001	1.05	1.02 – 1.08	0.001*
**Vaccination**												
First dose less than 21 days	Reference			Reference			Reference			Reference		
Holding the second dose*	0.94	0.91 – 0.97	0.001*	0.97	0.93 – 1.01	0.100	0.87	0.81 – 0.93	<0.001	0.94	0.91 – 0.97	<0.001*
Full vaccination	0.91	0.85 – 0.97	0.003*	0.98	0.92 – 1.04	0.459	0.84	0.74 – 0.95	0.007	0.94	0.88 – 0.99	0.032*
**Observations**		1142		692			450			1142		
**R^2**	-			0.192			0.237			0.186		
**AUC**	-			87.4%			85.4%			86.4%		
